# Chronic Intermittent Hypoxia and MASLD in Obstructive Sleep Apnea: A Critical Appraisal of Mechanistic and Causal Evidence

**DOI:** 10.3390/ijms27209372

**Published:** 2026-10-21

**Authors:** Georgiana-Mihaela Cebuc Simeanu, Ioana-Gabriela Dragne, Adina Mitrea, Diana Clenciu, Diana-Cristina Protasiewicz-Timofticiuc, Alina-Liliana Popa, Denisa-Maria Mitroi, Bianca-Florentina Pîrvu, Ion-Cristian Efrem, Adina Dorina Glodeanu, Adina-Andreea Mirea, Adela-Gabriela Ștefan, Maria-Magdalena Roșu, Theodora-Claudia Radu-Gheonea, Beatrice-Elena Vladu, Ana-Maria Efrem, Lorena-Anda Dijmarescu, Mircea-Catalin Fortofoiu

**Affiliations:** 1 Doctoral School, University of Medicine and Pharmacy, 200349 Craiova, Romania; georgiana.simeanu@umfcv.ro (G.-M.C.S.); gabriela.dragne@umfcv.ro (I.-G.D.); alina.popa@umfcv.ro (A.-L.P.); denisa_maria2@yahoo.com (D.-M.M.); bianca.pirvu@umfcv.ro (B.-F.P.); catalin.fortofoiu@umfcv.ro (M.-C.F.); 2 Department of Diabetes, Nutrition and Metabolic Diseases, Faculty of Medicine, University of Medicine and Pharmacy of Craiova, 200349 Craiova, Romania; adina.mitrea@umfcv.ro (A.M.); adela.firanescu@yahoo.com (A.-G.Ș.); theodora.gheonea@umfcv.ro (T.-C.R.-G.); 3 Department of Diabetes, Nutrition and Metabolic Diseases, Faculty of Midwives and Nursing, University of Medicine and Pharmacy of Craiova, 200349 Craiova, Romania; maria.rosu@umfcv.ro; 4 Department of Internal Medicine—Internal Medicine, Faculty of Dentistry, University of Medicine and Pharmacy of Craiova, 200349 Craiova, Romania; cristian.efrem@umfcv.ro (I.-C.E.); glodeanuadina@yahoo.com (A.D.G.); 5 Department of Oro-Dental Prevention and Oral Health, Faculty of Dentistry, University of Medicine and Pharmacy of Craiova, 200349 Craiova, Romania; adina.mirea@umfcv.ro; 6 Research Centre in Dental Medicine, University of Medicine and Pharmacy of Craiova, 200349 Craiova, Romania; 7 Faculty of Medicine, University of Medicine and Pharmacy of Craiova, 200349 Craiova, Romania; beatricevladu75@gmail.com; 8 Faculty of Medicine, University of Medicine and Pharmacy “Iuliu Haţieganu” Cluj-Napoca, 400012 Cluj-Napoca, Romania; anamaref23@gmail.com; 9 Department of Obstetrics-Gynecology, Faculty of Medicine, University of Medicine and Pharmacy of Craiova, 200349 Craiova, Romania; lorenadijmarescu@yahoo.com; 10 Department of Medical Semiology, Faculty of Medicine, University of Medicine and Pharmacy of Craiova, 200349 Craiova, Romania

**Keywords:** obstructive sleep apnea, metabolic dysfunction-associated steatotic liver disease, chronic intermittent hypoxia, insulin resistance, hepatic fibrosis, sleep fragmentation, causal inference, CPAP, GLP-1 receptor agonists

## Abstract

Obstructive sleep apnea (OSA) and metabolic dysfunction-associated steatotic liver disease (MASLD) frequently coexist and share a common cardiometabolic substrate through a shared cardiometabolic substrate. This narrative review critically appraises the epidemiological, experimental, and interventional evidence proposed to link chronic intermittent hypoxia (CIH) and sleep fragmentation to hepatic steatosis, steatohepatitis, and fibrosis. We summarize the candidate mechanisms, including mitochondrial oxidative stress, hypoxia-inducible factor 1-alpha signaling, sympathetically driven lipolysis, and Kupffer and stellate cell activation and then examine the limits of the evidence supporting them: hypoxia-induced hypophagia and the scarcity of pair-fed controls in rodent models, species differences in the physiological response to hypoxia, publication bias, common-cause confounding by age and systemic inflammation, and the rarity of progressive liver disease in lean patients with anatomically driven OSA. We further review non-invasive diagnostic tools, polysomnographic hypoxemia indices, transient elastography, METS-IR, FIB-4, and emerging biomarkers, such as omentin-1 and follistatin-like 1, that enable bidirectional screening between sleep and hepatology clinics. Finally, we discuss therapeutic strategies, including continuous positive airway pressure, structured weight loss, and incretin-based pharmacotherapy (GLP-1 and dual GIP/GLP-1 receptor agonists), highlighting recent phase 3 evidence for tirzepatide in OSA and semaglutide in metabolic dysfunction-associated steatohepatitis. Current evidence supports CIH as a context-dependent amplifier of liver injury in patients with pre-existing metabolic dysfunction rather than as an established independent cause of MASH. This interpretation still justifies integrated screening and treatment pathways, while identifying the study designs needed to test causality directly.

## 1. Introduction

A growing body of evidence points to a bidirectional relationship between obstructive sleep apnea (OSA) and metabolic dysfunction-associated steatotic liver disease (MASLD). This entity has replaced the older diagnostic label of non-alcoholic fatty liver disease (NAFLD) [1,2]. Clinically, this overlap is substantial: in nationally representative cross-sectional data, MASLD was present in 51.4% of adults at high risk of OSA on the STOP-Bang questionnaire, rising to 68.3% among those with obesity [3,4]. Mechanistically, much of the literature attributes this connection to chronic intermittent hypoxia (CIH), the physiological hallmark of recurrent upper-airway obstruction during sleep, which has been proposed to generate oxidative stress, amplify inflammatory signaling, and worsen insulin resistance [5,6]. However, this proposition rests largely on experimental models and cross-sectional clinical associations, and OSA also involves sleep fragmentation, sympathetic arousal, and circadian disruption, which may contribute independently to metabolic liver injury.

A parallel line of research suggests that the gut–liver axis may also play a role in this comorbidity. The intestinal microbiota functions as an active metabolic and immune interface, and disruption of its normal composition, termed dysbiosis, has been implicated in obesity, insulin resistance, and several metabolic diseases. In the specific context of OSA, the recurring oxygen fluctuations characteristic of the disorder appear capable of reshaping both gut bacterial communities and their metabolic output, with downstream effects on intestinal barrier integrity and circulating inflammatory mediators. At the epithelial level, cyclical desaturation and reoxygenation have been reported to alter tight-junction protein expression and increase paracellular permeability, allowing translocation of bacterial endotoxin into the portal circulation, where it engages Toll-like receptor 4 signalling on Kupffer cells and amplifies hepatic innate immune activation [7].

**Figure 1:**
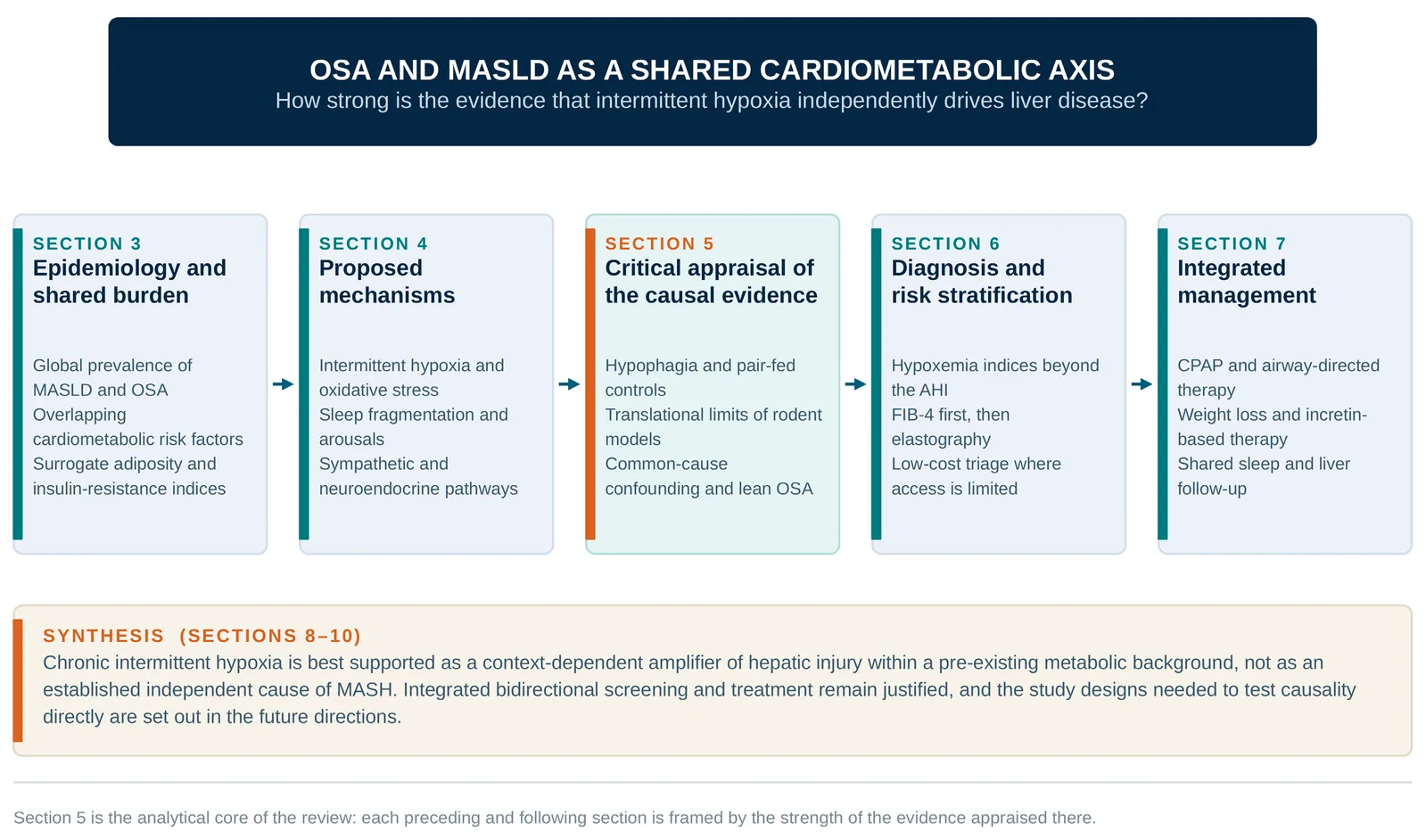
Structure and interpretive framework of the review. Each section is organized around a single question: whether chronic intermittent hypoxia independently drives liver disease in obstructive sleep apnea or amplifies it within a pre-existing metabolic background. Section 5 provides the critical appraisal on which the diagnostic and therapeutic sections are framed.

Rising global prevalence of both MASLD and sleep apnea makes an integrated clinical framework essential [5,6]. Whether nocturnal hypoxemia acts as an independent cause of MASLD progression, or instead amplifies liver injury only within a pre-existing metabolic background, remains unresolved. The distinction matters, because it determines whether correcting hypoxemia alone can be expected to modify the natural history of liver disease. In this review, we first summarize the epidemiological overlap and the proposed mechanisms and then critically appraise the evidence behind them, with particular attention to methodological confounders in animal models, translational limits, and common-cause bias in clinical studies. We then discuss the diagnostic and therapeutic implications of this appraisal. Figure 1 summarizes the structure of the review and the interpretive framework applied throughout.

## 2. Search Strategy

To evaluate the intersecting pathways of sleep-disordered breathing and progressive liver injury, we searched PubMed/MEDLINE, Embase, Scopus, and the Cochrane Library for peer-reviewed literature published from January 2000 to April 2026, using combinations of the following string, adapted to each database: (“sleep apnea, obstructive”[MeSH] OR “obstructive sleep apnea” OR “sleep-disordered breathing” OR “intermittent hypoxia” OR “sleep fragmentation”) AND (“non-alcoholic fatty liver disease”[MeSH] OR NAFLD OR MAFLD OR MASLD OR NASH OR MASH OR “hepatic steatosis” OR “liver fibrosis”).

We included original clinical studies, randomized controlled trials, experimental studies, systematic reviews, meta-analyses, and consensus guidelines published in English. We excluded case reports, conference abstracts, and studies of central sleep apnea, sustained (non-intermittent) hypoxia, or liver disease of alcoholic, viral, or autoimmune origin. Phase 3 trials published from 2024 onward (SURMOUNT-OSA and ESSENCE) were included because they provide the first large-scale randomized evidence on incretin-based therapy in each condition. To limit selective citation, we specifically searched for studies reporting neutral or negative findings, including sham-controlled CPAP trials, and for experimental studies reporting pair-fed control groups. Because this is a narrative rather than a systematic review, no formal risk-of-bias assessment was performed; instead, the strength of each line of evidence is appraised qualitatively in Section 5.

## 3. Epidemiology of MASLD and OSA

### 3.1. Nomenclature Change and Global Burden of MASLD

In 2023, a multi-society Delphi consensus convened by the American Association for the Study of Liver Diseases (AASLD), the European Association for the Study of the Liver (EASL), and several partner organizations formally retired the term NAFLD in favor of MASLD [1]. The change was intended to remove stigmatizing language tied to alcohol use and to anchor the diagnosis explicitly in cardiometabolic dysfunction. Rather than being defined by exclusion of alcohol consumption, MASLD is now defined positively around at least one cardiometabolic risk factor, obesity, type 2 diabetes, dyslipidemia, or hypertension [2].

MASLD is now the most common chronic liver disorder worldwide. A 2024 meta-analysis pooling 479 studies and more than 78 million participants across 38 countries estimated global prevalence at 30.2%, ranging regionally from 16.1% in Australia to 34% in South America [8]. Current epidemiological projections place today’s adult MASLD prevalence near 38%, with modeling suggesting this could exceed 55% by 2040; MASLD has also become the leading indication for liver transplantation among women and among patients with hepatocellular carcinoma in the United States [9]. Within OSA cohorts specifically, cardiometabolic burden is disproportionately high: obesity has been consistently identified as one of the strongest risk factors for OSA across global epidemiological data, alongside male sex, older age, and craniofacial predisposition [10]. Consistent with this shared risk-factor profile, a recent narrative synthesis of the relationship between metabolic syndrome and its major comorbidities concluded that OSA shows an independent association with the individual components of metabolic syndrome, including central obesity, dyslipidemia, and insulin resistance, situating OSA alongside MASLD as parallel downstream manifestations of the same underlying metabolic dysfunction [11].

### 3.2. Epidemiology of Obstructive Sleep Apnea

Pharyngeal collapse during sleep defines obstructive sleep apnea, which causes repeated airflow cessation, oxygen desaturation, and brief microarousals [12]. Worldwide, roughly 936 million adults aged 30–69 suffer from sleep apnea. Over 425 million meet criteria for moderate-to-severe disease requiring active medical treatment [13]. The highest case numbers cluster in China, the United States, Brazil, and India [13,14]. Community screening shows prevalence rates between 9% and 38% at an Apnea-Hypopnea Index threshold of 5 or more events per hour. Using a stricter cutoff of 15 or more events per hour drops prevalence to 6–17% (Table 1) [12,14]. Rates climb sharply with age, reaching up to 90% in elderly male cohorts [12]. A recent bibliometric analysis tracking publications from 2010 through 2024 documented a steep rise in research output, pointing to growing clinical interest in this comorbid pair [15].

OSA prevalence has grown by roughly 12% over the past decade, most rapidly in urbanizing regions of Asia and Latin America, tracking closely with rising rates of obesity, type 2 diabetes, and metabolic syndrome [14]. Established risk factors include male sex, older age, elevated body mass index, increased neck circumference, central adiposity, and craniofacial predisposition [16,17]. OSA also occurs at higher rates in patients already diagnosed with MASLD [3]. This co-occurrence is consistent with shared cardiometabolic pathways, but it does not by itself indicate the direction of the relationship or exclude common upstream causes (Section 5.4).

The strength of this association is illustrated by a systematic review of 28 studies, which found that OSA-related indicators were significantly correlated with NAFLD presence and severity in the large majority of included studies, although the apnea-hypopnea index alone proved an inadequate standalone predictor [3]. In a cohort of patients with severe obesity, mean nocturnal oxygen saturation, rather than BMI, sex, or the apnea-hypopnea index, emerged as the strongest independent predictor of FIB-4, a validated marker of advanced liver fibrosis, suggesting that the depth of nocturnal hypoxemia may be more informative than apnea frequency, although the cross-sectional design does not permit causal inference [16].

### 3.3. Shared Biomarkers and the Adipose–Liver Axis

Single laboratory markers rarely capture the full extent of metabolic risk in sleep apnea. To overcome this limitation, clinicians are increasingly relying on calculated insulin resistance scores such as METS-IR derived from fasting glucose, triglycerides, BMI, and HDL, which have been validated against insulin-resistance measures in general and metabolically at-risk populations [18]. Similarly, the Triglyceride-Glucose (TyG) index and its body-mass variation (TyG-BMI) act as strong surrogates for identifying advanced hepatic steatosis in sleep apnea patients [19]. Combining these blood-based panels with non-invasive imaging, such as the Hepatic Steatosis Index or transient-elastography-derived Controlled Attenuation Parameter (CAP), provides a direct measure of liver fat that has been shown to track with OSA severity [20]. Early integration of these surrogate markers helps medical teams spot silent metabolic deterioration well before irreversible tissue damage occurs [18,19,20]. 

Visceral fat acts as an active endocrine organ linking upper-airway collapse to liver damage. Omental and mesenteric depots dump pro-inflammatory signaling molecules directly into portal circulation, loading the liver with continuous cytokine stress [6,8]. In contrast to damaging proteins like TNF-α and IL-6, omentin-1 (intelectin-1) protects metabolic function. It enhances insulin sensitivity and nitric oxide synthase activity while suppressing inflammation. Pooled data from adult sleep apnea cohorts show that serum omentin-1 drops steadily as disease severity rises, falling in step with higher Apnea-Hypopnea Index values. These lower levels point to omentin-1 as a useful marker for obesity-related liver injury [21]. More recently, circulating follistatin-like 1 has shown a clear relationship with both disease severity and therapeutic response [22]. Signals from adipose and muscle tissue actively drive this systemic metabolic burden [6,22].

In a Romanian general-population cohort of over 2500 adults, the TyG index identified metabolic syndrome more accurately than competing formulas and retained independent predictive value after adjustment for age and METS-IR [18]. A meta-analysis of over 16,000 individuals further showed higher TyG values in patients with OSA than in controls [23]. These low-cost indices can therefore support metabolic risk stratification at sleep-clinic intake, although they reflect the shared metabolic background rather than any hypoxia-specific effect.

Reference values for these indices remain cohort-dependent rather than universally standardized. METS-IR values above 45 are commonly used to denote high metabolic risk, and TyG thresholds derived from Romanian general-population data identified metabolic syndrome with better discrimination than competing formulas [18], but cut-offs differ across ethnic groups and study designs, which limits their direct transfer between populations.

## 4. Pathophysiological Mechanisms: The Multi-Hit Convergence

### 4.1. Proposed Hypoxia-Related Mechanisms

The shift from the NAFLD to the MASLD framework reframes fatty liver disease as an inherently multi-system metabolic disorder. When OSA coexists with MASLD, disease progression is thought to accelerate through a “multi-hit” convergence of biomechanical, neuroendocrine, and cellular insults, a concept consistent with the broader multiple-parallel-hits model originally proposed for fatty liver disease [24]. Nutritional excess and sedentary behavior establish a baseline of simple steatosis, onto which the intermittent airway collapse of OSA appears to superimpose a distinct additional burden. In a rodent study, chronic intermittent hypoxia was associated with hepatic oxidative stress, mitochondrial dysfunction, and inflammatory changes, and these effects were more pronounced when hypoxia was combined with a high-fat diet [25]. This pattern is compatible with hypoxia adding a pathogenic layer to diet-driven fat accumulation; however, as discussed in Section 5.2, the absence of pair-fed controls in most such studies limits the attribution of these changes specifically to hypoxia.

**Figure 2:**
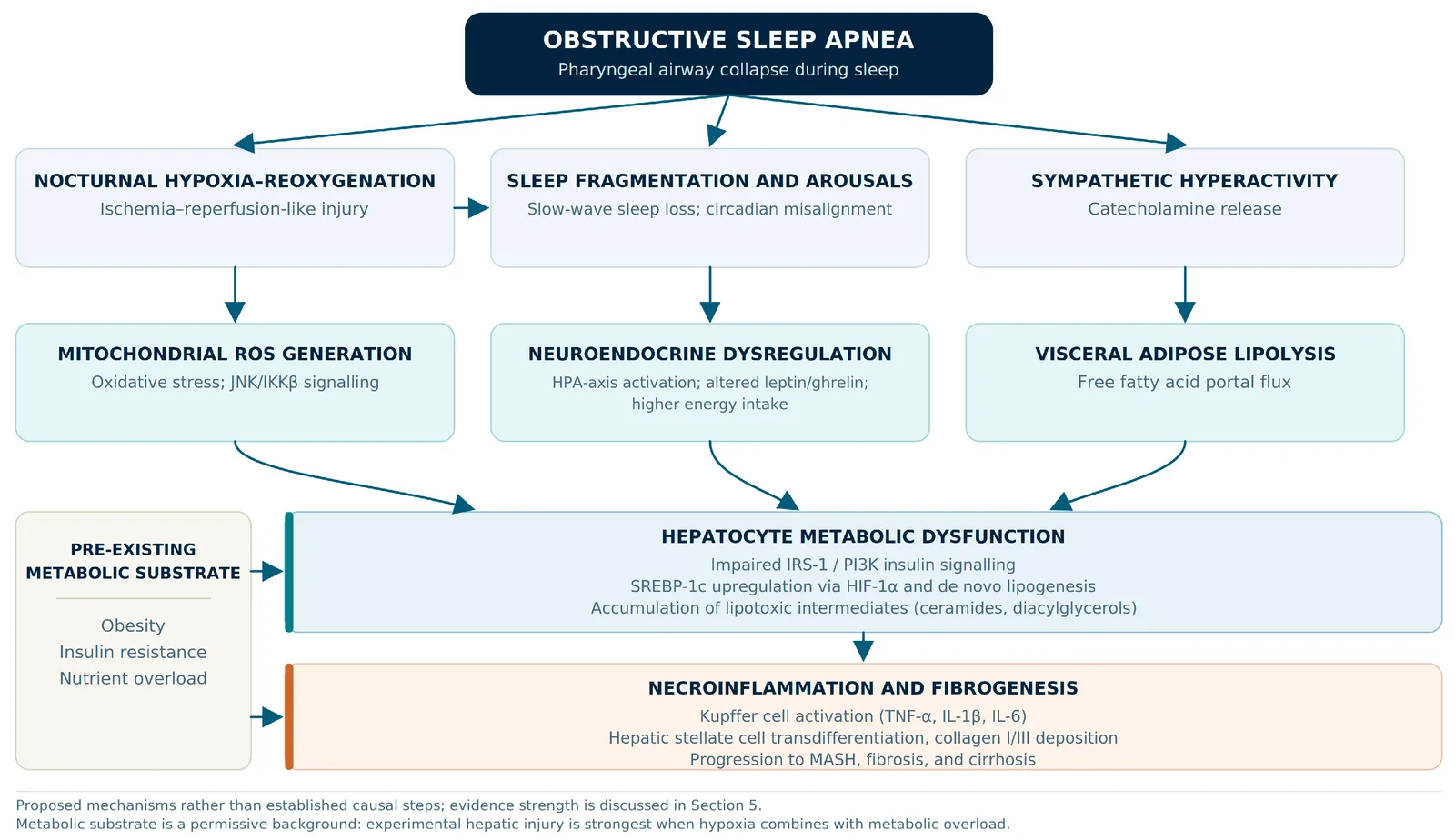
Proposed pathogenic pathways linking obstructive sleep apnea to MASLD progression. Intermittent hypoxia, sleep fragmentation, and sympathetic activation converge on hepatocyte metabolic dysfunction and necroinflammation, with pre-existing metabolic dysfunction acting as a permissive background. The pathways shown are proposed rather than established; their evidential status is appraised in Section 5.

Unlike the sustained hypoxia seen in chronic obstructive pulmonary disease or high-altitude exposure, OSA is defined by rapid cycles of desaturation and reoxygenation that have been likened to ischemia–reperfusion injury, an analogy derived mainly from experimental settings with more severe hypoxic exposure than typical clinical OSA. During apneic events, mitochondrial electron transport stalls; on airway reopening, an abrupt surge of electrons generates high concentrations of superoxide and hydrogen peroxide that overwhelm hepatic glutathione and superoxide dismutase reserves [25]. This oxidative burden intersects with HIF-1α in a notably dual manner: HIF-1α is understood to normally restrain ferroptosis, an iron-dependent form of hepatocyte death driven by inactivation of the lipid-repair enzyme glutathione peroxidase 4 and the resulting build-up of lipid hydroperoxides, partly by sustaining expression of the cystine transporter SLC7A11; when hypoxic signaling is disrupted or overwhelmed, however, mitochondrial ROS production rises further, adding a distinct, iron-dependent route of hepatocyte injury alongside the classical inflammatory pathways described above [26] (Figure 2).

Downstream of visceral fat, repetitive hypoxemia and hypercapnia drive sympathetic outflow and catecholamine release during sleep. The sympathetic nervous system is a principal regulator of adipocyte lipolysis, and both clinical OSA and experimental models of intermittent hypoxia have been shown to raise circulating catecholamine levels, which stimulate adipocyte β3-adrenergic receptors and accelerate the breakdown of stored triglycerides into free fatty acids and glycerol. Because obesity and insulin resistance already increase adipocyte mass and hormone-sensitive lipase activity, this surge in lipolytic tone, driven by both hypoxemia and arousal-related sympathetic activation (Section 4.2), may compound a pre-existing tendency toward excess fatty-acid output. The resulting flux of free fatty acids drains directly through the portal vein into the liver, delivering a substrate load the organ is poorly equipped to buffer during recurrent hypoxic stress. This sympathetically mediated route is considered one of several interconnected systemic and hepatic mechanisms by which intermittent hypoxia is thought to worsen fatty liver disease alongside direct upregulation of hepatic lipid-biosynthetic pathways, induction of oxidative stress within the liver, and hypoxia-driven transcriptional changes together forming a self-reinforcing cycle linking nocturnal airway obstruction to progressive hepatic lipid accumulation [6]. Hepatocytes attempt to clear this lipid load through mitochondrial β-oxidation and re-esterification, but hypoxia-induced suppression of mitochondrial respiratory complexes limits fatty acid oxidation, diverting the surplus into toxic diacylglycerols and ceramides that trigger endoplasmic reticulum stress and caspase-dependent hepatocyte apoptosis [27,28].

At the transcriptional level, chronic hypoxia inhibits the prolyl hydroxylase enzymes that normally target hypoxia-inducible factor (HIF)-α subunits for degradation, allowing HIF-1α and HIF-2α to accumulate and translocate to the nucleus. In hepatocytes, HIF-1α cooperates with liver X receptor alpha to upregulate SREBP-1c, the master regulator of de novo lipogenesis, increasing expression of fatty acid synthase and acetyl-CoA carboxylase in an already fat-overloaded liver [27,29], though a recent review cautions that HIF-1α’s net metabolic effect appears context- and model-dependent, sometimes preserving rather than worsening insulin sensitivity, underscoring that hypoxia signaling in MASLD is not uniformly pathogenic [29].

The transition from benign steatosis to steatohepatitis is driven substantially by resident non-parenchymal cells. Hepatic macrophages (Kupffer cells) sense hypoxic and lipotoxic stress and polarize toward a pro-inflammatory M1 phenotype, assembling the NLRP3 inflammasome and secreting IL-1β, IL-6, and TNF-α [30,31], altering sinusoidal endothelial permeability and recruiting circulating neutrophils and monocytes into the liver lobule [31]. Paracrine signaling from injured hepatocytes and activated Kupffer cells subsequently activates hepatic stellate cells via TGF-β1 and reactive oxygen species, driving their transdifferentiation into collagen-secreting myofibroblasts that establish the fibrous septa characteristic of advanced disease [24,31]. This overall sequence is consistent with the long-standing “multiple parallel hits” model of fatty liver disease progression [24]. Early animal work reported that intermittent hypoxia induced hepatitis in diet-primed mice [32], although, like most studies of that period, it did not include pair-fed controls. In humans, a prospective clinicopathological study of 582 bariatric surgery patients undergoing intraoperative liver biopsy found that OSA presence and severity were associated with a higher likelihood of steatohepatitis and significant fibrosis [33]. Because all participants had severe obesity, this study supports a contribution of OSA within an obese background, but it cannot determine whether hypoxemia would have the same effect in its absence (Table 2).

Experimental data suggest that intermittent hypoxia may also induce epigenetic reprogramming. Without changing the DNA sequence itself, chronic intermittent hypoxia alters gene expression through histone modification, DNA methylation, and non-coding RNA shifts including microRNAs and circular RNAs [34]. These regulatory marks endure long after individual apneic episodes end [34]. If confirmed in humans, such persistence could help explain why liver injury may progress despite fluctuations in nightly apnea counts [34]. Because several of these microRNAs circulate stably in peripheral blood, they serve as promising, minimally invasive markers to complement standard polysomnography and biochemical panels [34].

### 4.2. Sleep Fragmentation as a Distinct Pathogenic Component

OSA is not only a disorder of intermittent hypoxia; each obstructive event typically terminates in a cortical arousal, producing fragmented sleep with reduced slow-wave sleep. Attributing liver injury almost entirely to CIH therefore overlooks a second component that can act independently. In healthy volunteers, experimental fragmentation of sleep without any hypoxic exposure reduced insulin sensitivity and glucose tolerance [35]. Short or disrupted sleep is also associated with lower leptin and higher ghrelin concentrations and increased appetite [36], providing a behavioral route to positive energy balance and hepatic fat accumulation that does not depend on desaturation. In mice, sleep fragmentation in normoxia promoted NADPH oxidase 2-dependent visceral adipose tissue inflammation and insulin resistance [37], mirroring several effects usually ascribed to CIH. Arousals themselves elicit sympathetic surges and activation of the hypothalamic–pituitary–adrenal axis, so part of the lipolytic and glucocorticoid burden discussed above may reflect fragmentation rather than hypoxemia. Finally, disrupted sleep–wake timing may misalign hepatic clock genes that regulate de novo lipogenesis and fatty acid oxidation. Because hypoxemia and fragmentation are tightly coupled in clinical OSA, their relative contributions cannot currently be separated in human studies, and future work should report arousal-based indices alongside hypoxemic metrics. Both components are represented in Figure 2.

## 5. Critical Appraisal of the Causal Evidence

### 5.1. Origin and Propagation of the CIH–MASH Hypothesis

The proposition that CIH directly drives MASH originated in a small number of experimental studies published largely between 2007 and 2012, most notably murine models in which intermittent hypoxia was superimposed on a high-fat diet [6,32]. These early findings were hypothesis-generating. Over the following decade, however, the hypothesis was restated across numerous secondary reviews, and its status gradually shifted from plausible mechanism to presumed consensus without a proportional increase in evidence from designs capable of isolating the effect of hypoxia. The frequency with which a mechanism is cited should therefore not be mistaken for the rigor with which it has been tested.

### 5.2. Methodological Confounders in Rodent Models: Hypoxia-Induced Hypophagia

Rodents housed in hypoxic chambers consistently eat less than normoxic controls, an effect related to stress and to hypoxia-induced changes in appetite regulation. Because reduced food intake itself alters hepatic lipid handling, inflammatory tone, and insulin sensitivity, differences between CIH-exposed animals and ad libitum-fed normoxic controls cannot be attributed specifically to hypoxia. Pair-fed control groups, in which normoxic animals receive the same amount of food as hypoxic animals, are required to isolate the hypoxic effect, yet they remain uncommon, and several of the experimental studies cited in Section 4 did not report them [25,30,32]. Recent work that addressed dietary confounding found that CIH aggravated diet-induced steatohepatitis, fibrosis, and hepatic DNA damage through selective autophagic degradation of the DNA repair protein Eepd1 [38]. Such data indicate that a hypoxia-specific hepatic effect can persist once food intake is controlled, but they derive from a single model and require independent replication. It is also notable that hypoxic exposure without dietary priming generally produces little steatosis and that hepatic injury emerges mainly in combination with a high-fat diet, which is more consistent with an interaction between hypoxia and metabolic load than with an independent causal effect.

### 5.3. Translational Gaps Between Rodent Models and Human OSA

Rodents respond to hypoxia with rapid reductions in body temperature and metabolic rate, an adaptive hypometabolic response that has no clear equivalent during nocturnal desaturation in humans and that itself alters hepatic energy metabolism. Experimental protocols typically deliver hypoxic cycles at frequencies and nadirs corresponding to the most severe end of the clinical spectrum, in young, genetically homogeneous, and predominantly male animals, over weeks rather than decades. Rodent lipoprotein metabolism and basal metabolic rate also differ substantially from those of humans. In addition, neutral or negative findings from basic models are less likely to be published, so the available experimental literature probably overstates the consistency and magnitude of hypoxia-induced liver injury. These differences do not invalidate animal data, but they limit their direct transfer to human pathology.

### 5.4. Common-Cause Confounding, Reverse Causation, and the Lean OSA Argument

The frequent co-occurrence of OSA and MASH does not establish a direct causal link. Aging and systemic low-grade inflammation can independently promote upper-airway collapsibility and hepatic metabolic decline, producing parallel correlations between the two conditions. Multivariable adjustment is limited by residual confounding, particularly when adiposity is captured by BMI rather than by visceral fat, and Mendelian randomization offers only limited help here, because genetic instruments for obstructive sleep apnea are few and weak, and many act through adiposity so that the exclusion-restriction assumption is unlikely to hold. Reverse causation is also plausible since visceral and pharyngeal fat deposition and insulin resistance may themselves worsen OSA. Clinical cohorts are further enriched for obesity, as in sleep-clinic and bariatric populations [33], which restricts the ability to separate hypoxic from metabolic effects.

A key logical test concerns lean patients whose OSA arises from anatomical obstruction, such as retrognathia or tonsillar hypertrophy. If CIH were an independent and sufficient cause of MASH, these patients should show an excess of progressive liver disease; available evidence does not indicate that they do. This inference must be qualified, because data in lean OSA are sparse and these patients may have a lower hypoxic burden, but it argues against extrapolating causal claims from cohorts in which CIH operates almost exclusively within a metabolic comorbidity background. Consistent with this, sham-controlled CPAP trials, which offer the most direct human test of the hypoxic hypothesis, have generally not demonstrated improvements in hepatic steatosis or fibrosis over short-term follow-up [39,40,41]. These null results may reflect insufficient duration or adherence, a modest effect size, or the absence of an independent effect; they cannot currently distinguish between these explanations.

### 5.5. Synthesis

**Figure 3:**
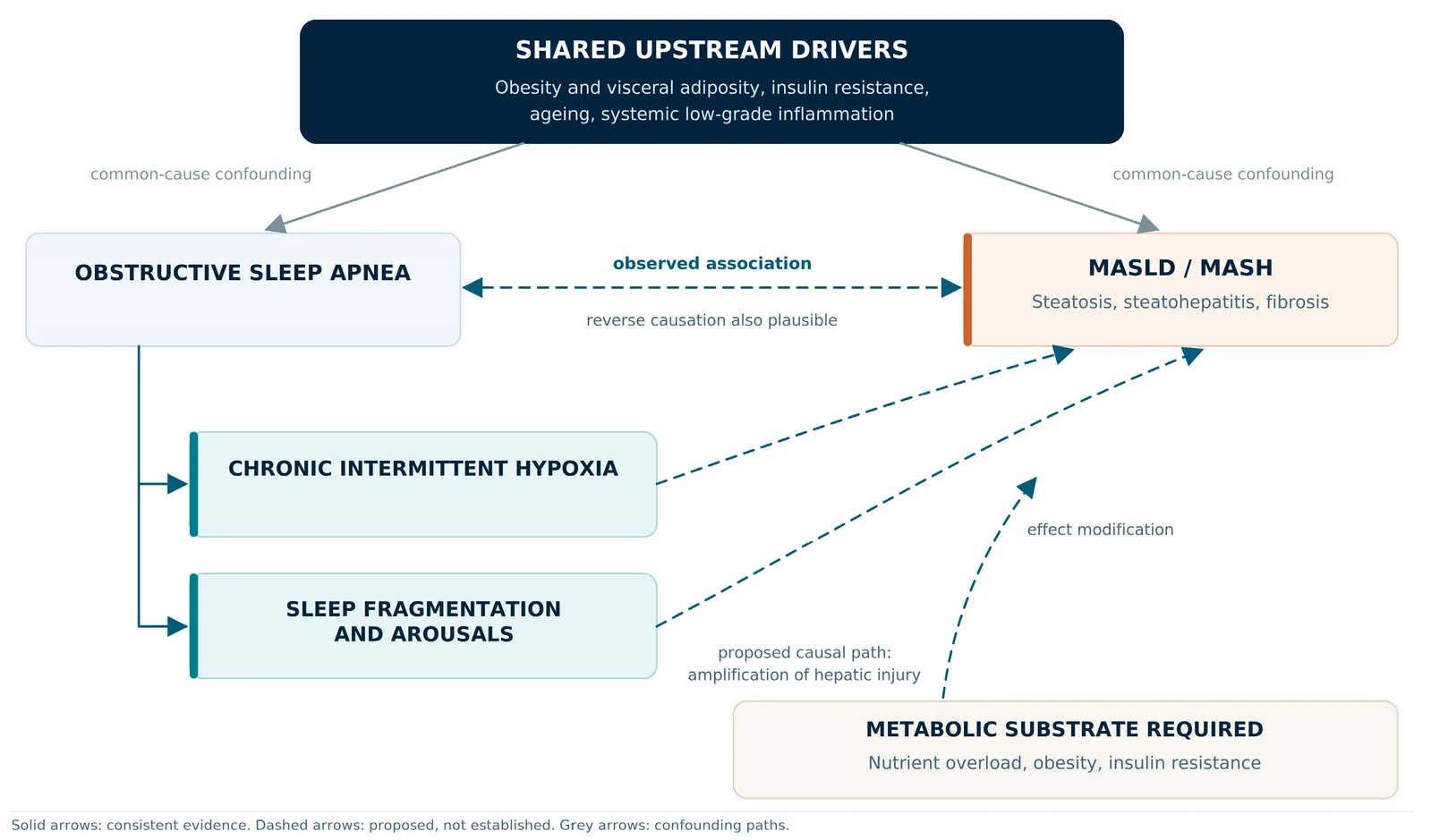
Competing explanations for the observed association between obstructive sleep apnea and MASLD/MASH. Shared upstream drivers, including obesity and visceral adiposity, insulin resistance, ageing, and systemic low-grade inflammation, can independently promote upper-airway collapsibility and hepatic metabolic decline, generating parallel correlations that are difficult to remove by multivariable adjustment. The association is also bidirectional, since visceral and pharyngeal fat deposition and insulin resistance may worsen sleep-disordered breathing. Chronic intermittent hypoxia and sleep fragmentation are the two candidate causal components of OSA, but current evidence supports them mainly as amplifiers of hepatic injury conditional on a pre-existing metabolic substrate rather than as independent causes. Solid arrows indicate relationships supported by consistent evidence, dashed arrows proposed but not established relationships, and grey arrows confounding paths.

Taken together, the evidence is most consistent with CIH acting as a context-dependent amplifier of liver injury in the presence of obesity and insulin resistance, rather than as an established independent cause of MASH (Figure 3). Table 3 summarizes the strength and main limitations of each line of evidence.

## 6. Diagnostic Modalities and Clinical Biomarkers

Managing comorbid OSA and MASLD presents a significant diagnostic hurdle, as most sleep apnea patients developing steatohepatitis remain completely asymptomatic until advancing toward end-stage liver injury. Standard liver panels offer inadequate sensitivity for early detection; routine transaminases (ALT/AST) frequently remain within normal reference ranges in over half of individuals with biopsy-confirmed steatohepatitis or significant fibrosis. Consequently, relying solely on serum transaminases misses a substantial proportion of progressive liver disease. To bypass these limitations, clinical protocols favor tiered screening approaches that begin with simple blood-based metrics like the FIB-4 index. When initial scores indicate elevated risk, clinicians use vibration-controlled transient elastography or quantitative MRI techniques to measure liver stiffness and fat accumulation. This step-by-step strategy filters out low-risk cases early. It also spots candidates for specialized hepatology care well before irreversible structural damage takes hold [42,43].

Polysomnography (PSG) remains the diagnostic benchmark for grading sleep apnea severity. Yet, the standard Apnea-Hypopnea Index often fails to capture the full extent of metabolic liver injury. Nocturnal hypoxemia metrics, specifically the Oxygen Desaturation Index (ODI), sleep time spent with SpO2 below 90% (T90), and minimum oxygen saturation, appear more closely associated with liver disease severity than AHI alone. In cross-sectional analyses, prolonged desaturation was associated with higher odds of advanced fibrosis after adjustment for body mass index or visceral fat [20,44], although residual confounding cannot be excluded.

To stratify this risk, clinicians now rely on non-invasive imaging instead of traditional liver biopsy [42]. Vibration-controlled transient elastography (VCTE) measures two distinct tissue properties in a single session: the Controlled Attenuation Parameter (CAP) for fat accumulation and Liver Stiffness Measurement (LSM) for fibrotic tissue [20,43]. In sleep-clinic populations, VCTE detects significant steatosis (CAP > 248 dB/m) in a large proportion of individuals with moderate-to-severe OSA [42]. In regression models, the frequency of nocturnal desaturations was associated with higher liver stiffness values, although residual confounding by adiposity cannot be excluded. This helps pinpoint high-risk individuals who require timely hepatology referral [20,44] (Table 4).

Standard fasting insulin measurements and HOMA-IR frequently fail to capture the peripheral metabolic damage caused by nocturnal hypoxemia. To overcome this diagnostic limitation, clinicians rely on composite glycemic-lipid panels. Composite indices such as METS-IR and the Triglyceride-Glucose (TyG) index capture peripheral metabolic dysfunction more reliably than fasting insulin alone, and TyG values are consistently higher in patients with OSA than in controls [18,23]. In parallel, indicators of visceral fat burden such as the Visceral Adiposity Index (VAI), the Lipid Accumulation Product (LAP), and specialized visceral fat scores mirror both OSA severity and progressive liver pathology [45,46]. These indices mainly capture the shared metabolic background and do not by themselves isolate a hypoxia-specific contribution [46,47].

For fibrosis screening, simple laboratory calculations provide a rapid first-line filter. The FIB-4 index, calculated from age, AST, ALT, and platelet count, excludes advanced fibrosis at scores below 1.30 with a negative predictive value above 90%. This cutoff allows sleep clinics to rule out severe disease early, directing only high-risk cases toward elastography and specialized hepatology care [42].

Beyond the adiposity-based indices discussed above, simple lipid ratios have also been explored as low-cost predictors of fatty liver disease in patients with type 2 diabetes, a population that overlaps substantially with the OSA-MASLD cohort. In a cross-sectional study of adults with type 2 diabetes, roughly half met ultrasound criteria for fatty liver disease. After adjustments for age, sex, diabetes duration, lipid-lowering therapy, and smoking status, elevated triglycerides, a higher atherogenic index of plasma, an increased non-HDL-to-HDL cholesterol ratio, and elevated HOMA-IR each remained independently associated with the disease, with specific cutoff values proposed for each [48]. Because these measures require only a routine fasting lipid panel and glucose or insulin values, they may offer a practical complement to the sleep- and hepatology-specific tools discussed above, particularly in settings where transient elastography or advanced imaging is not readily accessible.

Where specialized resources are available, magnetic resonance imaging proton density fat fraction (MRI-PDFF) quantifies liver fat with near-histological accuracy. A pooled analysis of nearly 3000 participants confirms that MRI-PDFF accurately distinguishes steatotic from non-steatotic tissue, although its discrimination slightly decreases at extreme fat concentrations [49]. Head-to-head comparisons show that MRI techniques consistently outperform standard ultrasound in detecting early, low-grade fat accumulation. High equipment costs and limited access restrict its routine use in sleep clinics [49,50]. As a result, MRI-PDFF serves primarily as the reference standard for validating accessible point-of-care tools such as ultrasound-derived CAP and circulating lipid-glycemic indices [49]. Integrating these routine biomarkers with non-invasive imaging creates a practical roadmap for staging fat accumulation, active liver inflammation, and fibrotic scarring. This multi-step approach reduces unnecessary biopsies while capturing progressive disease before permanent structural damage occurs [51].

## 7. Therapeutic Strategies and Clinical Management

### 7.1. Airway-Directed and Metabolic Therapy

Managing coexisting OSA and MASLD demands a joint strategy targeting both upper-airway collapse and systemic metabolic dysfunction. Treating only one organ system rarely resolves tissue damage when underlying metabolic drivers remain active [39,52]. Continuous positive airway pressure (CPAP) remains the primary treatment for moderate-to-severe sleep apnea. By acting as a pneumatic splint, CPAP prevents pharyngeal obstruction, restores nocturnal oxygenation, and blunts sympathetic nervous system hyperreactivity [39]. A meta-analysis, largely of observational studies, reported reductions in serum ALT and AST with CPAP use [53]; however, sham-controlled randomized trials have generally not confirmed improvements in liver enzymes, steatosis, or fibrosis [39,40,41]. In an observational cohort followed for up to four years, sustained CPAP adherence was associated with improvements in insulin resistance and hepatic fat [52]; however, uncontrolled designs of this type cannot separate the effect of CPAP from concurrent weight change or adherence-related health behaviors.

**Figure 4:**
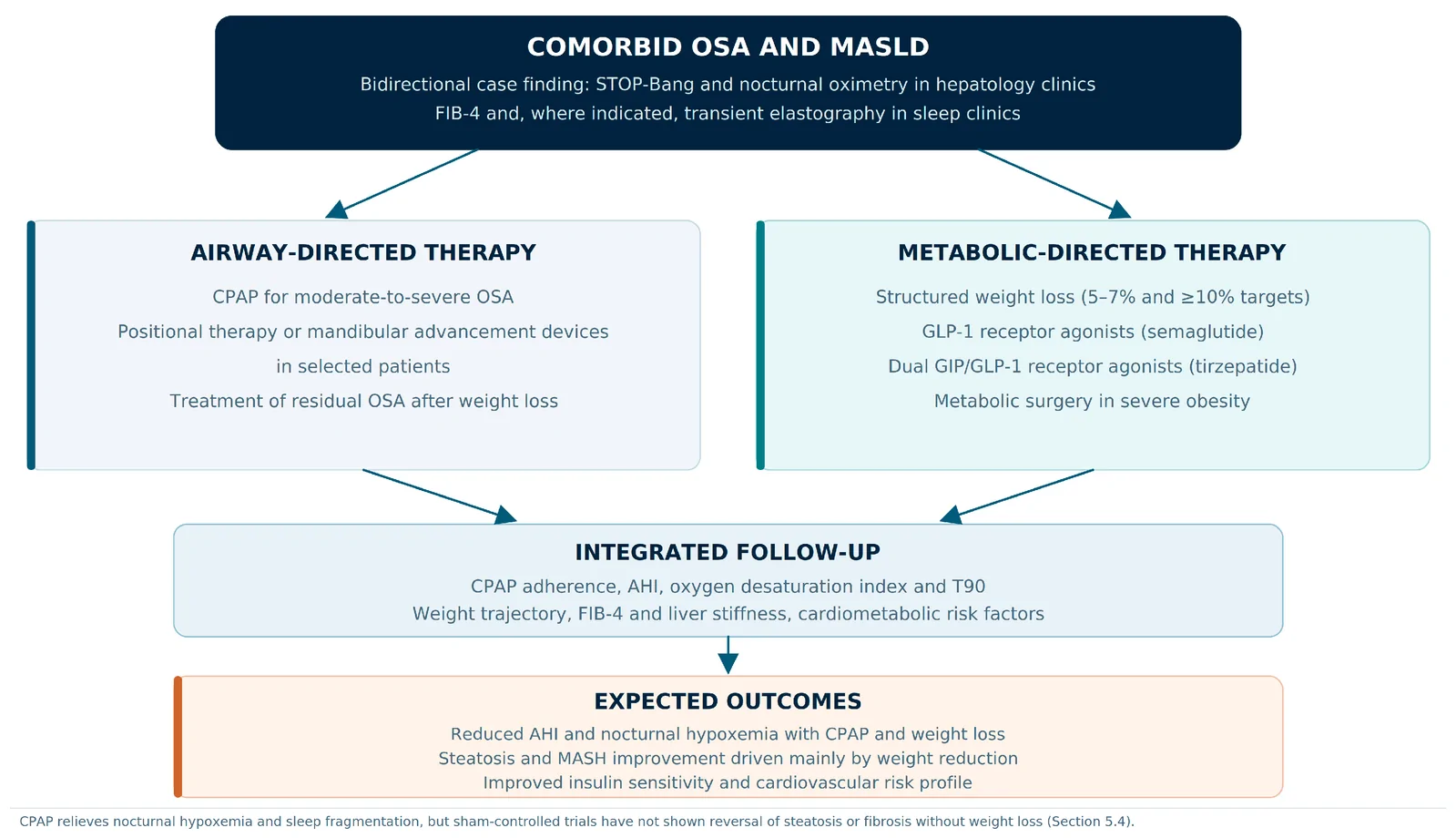
Integrated management framework for comorbid obstructive sleep apnea and MASLD. Bidirectional case finding is followed by parallel airway-directed and metabolic-directed treatment, with shared follow-up metrics covering both sleep and liver endpoints. CPAP relieves nocturnal hypoxemia and sleep fragmentation, but sham-controlled trials have not demonstrated reversal of steatosis or fibrosis in the absence of weight loss (Section 5.4).

Yet, mechanical airflow has clear limits. Although eliminating nocturnal hypoxia would be expected to reduce oxidative stress, CPAP alone rarely reverses established liver fibrosis without concurrent weight loss [54]. Patients with progressive MASLD typically suffer from ongoing nutrient overload and visceral adiposity that oxygen restoration cannot fix on its own [52,54]. Because of this gap, clearing nocturnal airway blockages must be paired with structured lifestyle changes, caloric restriction, or metabolic pharmacotherapy to achieve true histological recovery [39,54] (Figure 4).

Weight reduction remains the most effective foundational treatment for both conditions. A total body weight loss of 5–7% reduces hepatic steatosis and snoring severity, while losses of 10% or more are associated with histological resolution of steatohepatitis in up to 90% of patients and fibrosis regression in over 45% [55]; comparable weight loss also reduces fat deposition in the lateral pharyngeal walls and tongue base, with corresponding reductions in the apnea-hypopnea index.

Incretin-based pharmacotherapies have meaningfully changed the management of comorbid patients with obesity. In the phase 3 SURMOUNT-OSA program, once-weekly tirzepatide, a dual GIP/GLP-1 receptor agonist, reduced AHI substantially compared with placebo in adults with obesity and moderate-to-severe OSA [56], and prespecified secondary analyses published in 2026 further showed that tirzepatide improved a broad panel of cardiometabolic risk measures, with mediation analyses attributing much of the benefit to reductions in body weight, AHI, and hypoxic burden [57]. On the hepatology side, the phase 3 ESSENCE trial demonstrated that once-weekly semaglutide 2.4 mg produced significantly higher rates of steatohepatitis resolution without fibrosis worsening (62.9% vs. 34.3% with placebo) and significantly greater fibrosis improvement without worsening steatohepatitis (36.8% vs. 22.4%) at 72 weeks in patients with biopsy-confirmed MASH and stage F2–F3 fibrosis [58].

These parallel benefits appear to arise mainly through systemic metabolic effects. Incretin mimetics act centrally to promote satiety and delay gastric emptying, driving substantial and sustained weight loss that mobilizes visceral adipose tissue, reduces portal fatty-acid flux, and decreases pharyngeal fat volume; the accompanying improvements in insulin sensitivity and systemic inflammation follow largely from this weight loss [56,57,58,59]. Direct receptor-mediated effects on human hepatocytes should not be assumed. Although early work reported GLP-1 receptor expression in human hepatocytes, studies using extensively validated antibodies have not detected the receptor in normal or diseased human liver, and hepatocyte GIP receptor expression is likewise minimal, with GIP acting mainly on adipose tissue and the central nervous system [59,60,61]. The hepatic benefits of incretin-based therapies are therefore best interpreted as indirect consequences of weight loss, improved insulin sensitivity, and reduced adipose-tissue lipolysis, rather than as evidence of direct hepatocyte targeting [59]. Importantly, because these agents improve both AHI and liver histology largely through weight loss, their benefits cannot be used to infer that hypoxemia itself drives liver disease; rather, they highlight the shared metabolic substrate. These interacting pathways support multidisciplinary care: patients presenting to sleep clinics with moderate-to-severe OSA and metabolic syndrome should undergo baseline liver screening with FIB-4, while hepatology clinics evaluating MASLD patients should routinely apply validated OSA screening tools such as STOP-Bang [3,42]. Identifying coexisting OSA allows clinicians to combine CPAP with incretin pharmacotherapy or metabolic surgery, addressing both the hypoxic component and the metabolic basis of progressive liver disease [52,57].

For patients with severe obesity in whom lifestyle measures or pharmacotherapy prove insufficient, bariatric and metabolic surgery offers an additional route to substantial and durable weight loss, with corresponding benefits for both airway patency and liver fat content. Postoperative improvement or resolution of OSA is common, yet a recent narrative review cautioned that sleep-disordered breathing does not resolve completely in every patient and that residual disease after surgery is more likely among those with more severe baseline OSA, older age, male sex, and a smaller magnitude of postoperative weight loss. These observations argue against assuming that surgical weight loss alone eliminates the comorbid burden and instead support continued postoperative screening for persistent OSA and residual liver pathology [62].

Pharmacotherapy faces similar boundaries once advanced tissue scarring develops. In a randomized phase 2 trial involving patients with MASH-related compensated cirrhosis, weekly semaglutide 2.4 mg failed to meet its primary endpoint, showing no statistically significant improvement in liver fibrosis stage or cirrhosis reversal compared with placebo. Although semaglutide improved secondary cardiometabolic factors and body weight, the absence of histologic fibrosis regression highlights a clear therapeutic ceiling in established cirrhosis [63]. Combining surgical weight loss with targeted pharmacotherapy before irreversible structural damage takes hold likely offers the best pathway to protecting both respiratory function and long-term hepatic health [62,63].

Resmetirom differs from these approaches in being a liver-directed therapy. It is an oral, liver-targeted, selective thyroid hormone receptor-β agonist that increases hepatic fatty-acid oxidation and reduces lipotoxicity independently of weight loss. In the phase 3 MAESTRO-NASH trial, which enrolled patients with biopsy-confirmed noncirrhotic MASH and predominantly F2–F3 fibrosis, both the 80 mg and 100 mg doses achieved higher rates of MASH resolution without fibrosis worsening (25.9% and 29.9% vs. 9.7% with placebo) and of fibrosis improvement by at least one stage without worsening of the NAFLD activity score (24.2% and 25.9% vs. 14.2%) at 52 weeks [64]. On this basis, resmetirom received accelerated approval in the United States in 2024 and conditional marketing authorisation in the European Union in 2025 for adults with noncirrhotic MASH and moderate-to-advanced (F2–F3) fibrosis, and the EASL–EASD–EASO guidelines support its use in this population where approved [65]. Its place in the comorbid patient is therefore distinct: resmetirom acts on the hepatic disease process itself but does not reduce body weight and is not expected to improve OSA, whereas incretin-based therapies and metabolic surgery target the shared obesity substrate, and CPAP addresses nocturnal hypoxemia and sleep fragmentation. In patients with significant fibrosis, these strategies are complementary rather than interchangeable, although no trial has yet evaluated resmetirom specifically in patients with coexisting OSA.

### 7.2. Implementation in Resource-Limited Settings

Environmental exposures common in these settings, including household biomass combustion and ambient air pollution, may add to systemic inflammatory burden, although their specific contribution to the OSA–MASLD axis has not been quantified.

In low- and middle-income countries, access to polysomnography, transient elastography, CPAP, incretin-based therapies, and metabolic surgery is often limited, and both conditions may remain clinically silent until complications develop. In these settings, a pragmatic triage pathway combining validated questionnaires such as STOP-Bang, nocturnal oximetry to estimate hypoxemic burden, and simple serological indices such as FIB-4 and TyG may identify patients who warrant referral for further sleep and liver assessment. Fragmented referral pathways between primary care, pulmonology, and hepatology can further delay recognition of the combined phenotype. Such simplified pathways are useful for triage but cannot fully characterize OSA severity, hypoxic burden, or hepatic fibrosis, and their diagnostic performance in these populations requires prospective validation.

## 8. Future Directions

Future research should prioritize prospective longitudinal studies capable of determining whether obstructive sleep apnea independently accelerates the development and progression of metabolic dysfunction-associated steatotic liver disease after accounting for obesity, type 2 diabetes, visceral adiposity, dyslipidemia, medication use, and other cardiometabolic risk factors. Repeated assessment of both sleep-disordered breathing and liver disease would help clarify temporal relationships and identify the stage at which chronic intermittent hypoxia contributes most strongly to steatosis, steatohepatitis, and fibrosis.

A major priority is the standardization of phenotyping across studies. OSA severity should not be characterized exclusively by the apnea-hypopnea index, because conventional event frequency may inadequately represent the biological burden of nocturnal hypoxemia. Future studies should incorporate oxygen desaturation index, hypoxic burden, time spent below 90% oxygen saturation, nadir oxygen saturation, sleep fragmentation, and sympathetic activation. Similarly, hepatic outcomes should be assessed using standardized combinations of FIB-4, vibration-controlled transient elastography, controlled attenuation parameter, MRI-proton density fat fraction, and, when clinically justified, histological evaluation. Such harmonization would improve comparability across cohorts and facilitate the development of clinically useful risk thresholds.

Randomized controlled trials are also needed to determine whether effective OSA treatment modifies the natural history of MASLD. These studies should evaluate adequately monitored CPAP adherence over sufficiently long follow-up periods and use liver-specific endpoints rather than isolated changes in aminotransferase concentrations. Particular attention should be given to whether CPAP can reduce hepatic fat content, inflammatory activity, fibrosis progression, or cardiovascular risk independently of weight loss. Trials comparing CPAP alone with combined strategies involving structured weight reduction, GLP-1 receptor agonists, dual GIP/GLP-1 receptor agonists, metabolic surgery, or emerging antifibrotic therapies would be especially relevant.

Two study designs would address the causal question most directly. First, experimental studies should routinely include pair-fed normoxic controls, report body temperature and energy expenditure, and use hypoxic protocols calibrated to clinically representative severity, ideally in both sexes and in animals of different ages. Second, clinical studies should deliberately include lean patients with anatomically driven OSA as a natural contrast group, in whom the hepatic effect of intermittent hypoxia can be assessed in the relative absence of metabolic comorbidity. Separating hypoxemic indices from arousal-based measures of sleep fragmentation in the same cohorts would also clarify the relative contribution of each component.

Mechanistic investigations should further define how intermittent hypoxia interacts with visceral adipose tissue dysfunction, mitochondrial injury, ferroptosis, hepatic immune activation, stellate-cell signaling, gut dysbiosis, and epigenetic regulation. Multi-omics approaches integrating metabolomics, lipidomics, proteomics, transcriptomics, microbiome analysis, and circulating non-coding RNAs may identify molecular signatures that distinguish patients with metabolically uncomplicated OSA from those at high risk of progressive liver injury.

Implementation studies should determine the feasibility, cost-effectiveness, and clinical impact of bidirectional screening pathways between sleep and hepatology services. Together, these priorities would allow the hypothesis that intermittent hypoxia amplifies metabolic liver injury to be tested directly rather than inferred.

## 9. Limitations

This narrative review has several limitations. Although multiple databases were searched, the literature selection was not based on a registered systematic-review protocol, formal risk-of-bias assessment, or meta-analysis, and the restriction to English-language publications may have introduced selection and publication bias. Most available studies are observational and therefore cannot establish a causal relationship between OSA and MASLD, particularly because obesity, insulin resistance, diabetes, and visceral adiposity are major shared confounders. Considerable heterogeneity also exists in OSA definitions, hypoxemia indices, liver assessment methods, and fibrosis thresholds. In addition, much of the mechanistic evidence derives from experimental models, while clinical studies of CPAP and metabolic therapies frequently use short follow-up periods, variable adherence criteria, and surrogate rather than histological liver outcomes. Most experimental studies did not include pair-fed controls, and neutral preclinical findings are likely underrepresented in the published literature, so the mechanistic evidence summarized here may overstate the specificity and magnitude of hypoxia-induced liver injury. Finally, common-cause confounding by aging and systemic inflammation cannot be excluded in any of the available clinical designs.

## 10. Conclusions

Obstructive sleep apnea and metabolic dysfunction-associated steatotic liver disease frequently coexist, and several biologically plausible pathways, including oxidative stress, HIF-1α signaling, sympathetically driven lipolysis, sleep fragmentation, and hepatic immune activation, could link them. A critical appraisal of the evidence, however, indicates that chronic intermittent hypoxia is best regarded as a context-dependent amplifier of liver injury in patients with obesity and insulin resistance, rather than as an established independent cause of MASH. Rodent data are limited by hypoxia-induced hypophagia, species-specific hypoxic physiology, and publication bias; clinical associations are vulnerable to common-cause confounding and reverse causation; progressive liver disease appears uncommon in lean, anatomically driven OSA; and sham-controlled CPAP trials have not demonstrated a clear hepatic benefit.

Given the high prevalence of this comorbidity, siloed management by pulmonology or hepatology alone is no longer sufficient. Bidirectional screening using non-invasive tools, polysomnographic hypoxemia indices, transient elastography, and biomarker panels such as FIB-4 can help identify patients at risk for progressive liver disease. Optimal outcomes likely require combining mechanical airway stabilization via CPAP, which removes nocturnal hypoxemia and sleep fragmentation, with substantial weight reduction and incretin-based pharmacotherapy, which addresses systemic insulin resistance and visceral adiposity directly. Approaching OSA and MASLD as linked manifestations of one cardiometabolic syndrome, rather than as separate diseases, is central to reducing long-term hepatic and cardiovascular mortality in this growing patient population.

## Figures and Tables

**Table 1:** Global prevalence of obstructive sleep apnea by apnea-hypopnea index threshold. Prevalence ranges from community screening studies; burden estimates from literature-based global modelling.

Threshold	Community Prevalence	Estimated Global Burden (Adults 30–69 y)	Clinical Interpretation
AHI ≥ 5 events/h	9–38%	~936 million	Any sleep-disordered breathing; includes mild, often asymptomatic disease
AHI ≥ 15 events/h	6–17%	~425 million	Moderate-to-severe disease; threshold at which active treatment is usually indicated

**Table 2:** Shared pathophysiological mechanisms in obstructive sleep apnea and MASLD.

Mechanical/Cellular Pathway	Primary Physiological Driver	Direct Hepatic Manifestation
Chronic intermittent hypoxia	Repetitive arterial desaturation–reoxygenation cycles	Mitochondrial ROS production, lipid peroxidation, JNK/IKKβ-mediated insulin resistance
Sympathetic nervous system hyperactivity	Nocturnal catecholamine surge triggered by airway obstruction	Visceral adipose lipolysis, increased portal free fatty acid influx, ceramide/DAG accumulation
Transcriptional HIF-1α stabilization	Inhibition of oxygen-dependent prolyl hydroxylases	Synergistic upregulation of SREBP-1c, driving de novo lipogenesis
Kupffer cell and stellate cell activation	Paracrine cytokine signaling (TNF-α, IL-1β) and cell stress	Myofibroblast transdifferentiation, type I/III collagen deposition, lobular fibrogenesis
Sleep fragmentation and circadian disruption	Repeated cortical arousals, slow-wave sleep loss, misalignment of hepatic clock genes	HPA-axis activation, altered leptin/ghrelin balance and energy intake, adipose tissue inflammation, dysregulated hepatic lipogenesis

**Table 3:** Appraisal of the evidence linking OSA and chronic intermittent hypoxia to MASLD/MASH.

Line of Evidence	Main Finding	Principal Limitation	Contribution to Causal Inference
Rodent CIH models without pair-fed controls	Hepatic oxidative stress, inflammation and fibrosis, mainly in combination with a high-fat diet	Hypophagia and stress confound the hypoxic effect; publication bias	Low; supports biological plausibility only
Rodent CIH models with pair-fed controls	Hypoxia-specific aggravation of diet-induced steatohepatitis	Few studies; species differences in hypoxic physiology	Moderate for plausibility; limited for human causality
Cross-sectional clinical studies	Hypoxemic indices associated with steatosis and fibrosis markers	Residual confounding by adiposity; no temporality	Low
Biopsy cohorts in severe obesity	OSA severity associated with MASH and fibrosis	Uniformly obese populations; selection bias	Supports an effect within an obese background only
Mendelian randomization	Few studies; evidence insufficient	Weak OSA instruments; pleiotropy through adiposity	Low to moderate
Sham-controlled CPAP trials	Largely no effect on steatosis or fibrosis	Short follow-up; variable adherence	Does not support a large independent effect
Weight-loss and incretin trials	Parallel improvement in AHI and MASH	Cannot separate hypoxic from metabolic improvement	Supports a shared metabolic driver

Abbreviations: AHI, apnea-hypopnea index; CIH, chronic intermittent hypoxia; CPAP, continuous positive airway pressure; MASH, metabolic dysfunction-associated steatohepatitis; MASLD, metabolic dysfunction-associated steatotic liver disease; OSA, obstructive sleep apnea.

**Table 4:** Clinical biomarkers and diagnostic modalities in comorbid OSA and MASLD.

Diagnostic Tool/Modality	Targeted Metric	Diagnostic Cutoff/Reference Range	Clinical Application in Comorbid Cohorts
Polysomnography (PSG)—AHI & ODI	Apnea-Hypopnea Index, Oxygen Desaturation Index	Moderate: 15–29 events/h; Severe: ≥30 events/h	Quantifies airway obstruction; ODI and T90 associated with steatosis and fibrosis markers
VCTE (FibroScan)—CAP	Ultrasound attenuation (dB/m)	S1: >248 dB/m; S2: >268 dB/m; S3: >280 dB/m	Detects and grades hepatic fat content; frequently elevated in moderate-to-severe OSA [20]
VCTE (FibroScan)—LSM	Shear-wave velocity (kPa)	F2 (significant): >7.0 kPa; F3 (severe): >9.5 kPa; F4 (cirrhosis): >12.5 kPa	Identifies fibrotic transformation, associated with T90 and nocturnal nadir SpO2
METS-IR index	Glucose, triglycerides, BMI, HDL-C	High risk: score > 45.0	Outperforms HOMA-IR in predicting steatosis via peripheral lipid dysregulation
FIB-4 index	Age, AST, ALT, platelet count	Low risk: <1.30; High risk: >2.67	Primary outpatient filter to rule out advanced fibrosis in sleep clinics

## Data Availability

Not applicable.
